# Cannabis use patterns at the dawn of US cannabis reform

**DOI:** 10.1186/s42238-019-0003-z

**Published:** 2019-06-07

**Authors:** Navin Kumar, Cheneal Puljević, Jason Ferris, Adam Winstock, Monica J. Barratt

**Affiliations:** 1 Yale Institute for Network Science, Yale University, New Haven, CT USA; 2 Center for Empirical Research on Stratification and Inequality (CERSI), Yale University, New Haven, CT USA; 3 Department of Sociology, Yale University, New Haven, CT USA; 4 Centre for Health Service Research, University of Queensland, QLD, Brisbane, Australia; 5 University College London, London, UK; 6 Global Drug Survey Ltd, London, UK; 7 Drug Policy Modelling Program, National Drug and Alcohol Research Centre, UNSW Sydney, Sydney, NSW Australia; 8 National Drug Research Institute, Faculty of Health Sciences, Curtin University, Perth, WA Australia; 9 Behaviours and Health Risks Program, Burnet Institute, Melbourne, VIC Australia

**Keywords:** US legal Cannabis market, Cannabis preferences, Cannabis behaviors, Mode of consumption

## Abstract

In the United States (US), three in 10 cannabis users develop cannabis use disorder (CUD). Usage patterns in line with CUD may be associated with socio-economic disadvantage, and other negative effects. Thus, research on CUD is paramount. To provide understanding around CUD, it is necessary to detail granular cannabis usage preferences, as some risk from cannabis use may be mitigated through informed behavioral choices by users. We describe cannabis usage preferences among US Global Drug Survey (GDS) respondents, primarily young men. The cross-sectional web-based GDS (2017) was completed by 8345 US-resident respondents (median age = 23, Interquartile Range 19–32; % male = 75.48) who reported cannabis use. Of those who reported cannabis use in the past year, most (78%) reported consuming their first joint more than an hour after waking, and about half the sample (49%) had their last joint 1–2 h before bed. Cannabis was used for a median of 250 days in the last year (almost daily). Respondents spent a median of four hours a day stoned when cannabis was used. High potency herbal cannabis was the preferred variant by 62% of participants. We suggest that frequent use of cannabis may increase risk of health harms, and highlight the need to mitigate problematic use. With the rapidly developing US cannabis market, possibly problematic usage patterns may indicate potential for CUD especially within young men.

## Introduction

In the United States (US), three in 10 cannabis users develops cannabis use disorder (CUD) under DSM-IV guidelines (Hasin et al., 2015). When using DSM-5 guidelines, 19.5% of lifetime cannabis users met the criteria for CUD (Hasin et al., 2016). We define CUD as a problematic pattern of cannabis use leading to clinically significant impairment or distress as manifested by at least two of the markers of CUD, as defined by the DSM 5 (American Psychiatric Association, 2013). Usage patterns in line with CUD may be associated with socio-economic disadvantage, including unemployment or decreased financial stability (Brook et al., 2013). Research on CUD is paramount, to guide policy and interventions, especially with the rapid growth of US legal cannabis markets, given that states with legalized cannabis have greater rates of cannabis use and CUD (Cerdá et al., 2012). Cannabis may also provide some therapeutic benefits, for conditions such as multiple sclerosis and nausea (Grotenhermen & Müller-Vahl, 2012; Zajicek et al., 2012). There are also recommendations for lower risk use, such as avoiding early initiation of use, and using low-potency products (Fischer et al., 2017). In this vein, some of the risk from cannabis use may be mitigated through informed behavioral choices by users (Fischer et al., 2017).

Thus, to provide understanding around CUD, it is necessary to detail cannabis usage preferences. However, there is a paucity of research exploring preferences around US cannabis use. Past work has explored demographic characteristics and cannabis use preferences, but these generally use data prior to rapid legalization in recent years (Carliner et al., 2017; Compton et al., 2016; Hasin et al., 2017; Terry-McElrath et al., 2017). More recent data is key as additional jurisdictions rapidly legalize medical and recreational cannabis use, and the possibly associated changes in CUD. Moreover, while these studies report cannabis prevalence, primarily utilizing the National Survey on Drug Use and Health (NSDUH), National Epidemiologic Survey on Alcohol and Related Conditions (NESARC), and Monitoring the Future (MTF), they do not indicate nuanced data on usage preferences, such as time of use and preferred cannabis variants (edibles, resin etc.). For example, given the sheer range of cannabis products (Hutmacher, 2015), charting prevalence of cannabis is not sufficient if users have preferences for different products and some are more likely to contribute to CUD compared to others (Loflin & Earleywine, 2014). With the shifting US cannabis landscape, granular data on cannabis usage practices are key to pioneering policy and crafting future research. Using a USA-subset of a large cross-sectional online global survey, this paper describes a range of cannabis usage preferences, including time of first and last joint, quantities of use, and preferred forms of cannabis preparations. The survey questions we highlight are not in themselves the strongest markers of CUD, but understanding prevalence of cannabis use from a large sample may shed light on patterns of CUD.

## Methods

The Global Drug Survey (GDS) annually conducts anonymous, online surveys to investigate international trends in drug use, both legal and illicit. Data from GDS 2017, collected from November 15, 2016 to January 18, 2017, is utilized in this paper. The age and sex distributions of cannabis users who completed the GDS in Australia, the US, and Switzerland were similar to their respective countries’ demographic distributions in a household survey across the three countries (Barratt et al., 2017). When the GDS (2014) is compared to the similar NSDUH (2013) data, there are several key similarities. For example, regardless of age, men were more likely to report cannabis use compared to women. Both men and women typically demonstrate similar trends of a decreasing probability of lifetime and previous-year cannabis use with age. While the probability of ever using cannabis is greater in the GDS (2014) sample, the probability of using cannabis in the past year among lifetime users, and using within the past month among past-year users is comparable across GDS (2014) and NSDUH (2013) data. While non-response bias and volunteer bias may influence GDS samples, unmeasured confounders may affect data in household surveys (Keiding & Louis, 2016). Household surveys may underestimate the prevalence of illicit drug use due to stigma and other factors (Chalmers et al., 2016; Zhao et al., 2009). In addition, GDS is far cheaper given its higher response rate, compared to household surveys (Barratt et al., 2017). For example, in GDS (2014), 6419 users were surveyed to recruit 3879 past-month cannabis users. In comparison, the NSDUH (2013) surveyed 43,465 to recruit 5664. Thus, the GDS is an effective way of gaining a nuanced understanding of stigmatized behaviors, if it is not used to estimate drug prevalence of the general population (Barratt et al., 2017). Sample representativeness may only be necessary when exploring research questions about population prevalence estimates (Barratt et al., 2017), and the GDS is thus appropriate to provide insight about US cannabis usage preferences within specific samples such as young males.

The survey was actively promoted on social media platforms, such as Twitter, Facebook, and through media partners, such as, Mixmag and The Guardian (USA). All respondents confirmed they were 16+ years and provided informed consent. The study received institutional review board (IRB) approval from The Psychiatry, Nursing and Midwives Ethics subcommittee at Kings College, London (141/02), The University of Queensland (No: 2017001452) and The University of New South Wales (HREC HC17769). Analyses were first restricted to US-based respondents. Responses were included only if individuals indicated use of cannabis in the last 12 months, through all forms of administration, such as smoking, eating or vaporizing. The measures described in this paper include demographic characteristics, whether cannabis was mixed with tobacco in the last year, time of first joint, amount of cannabis used per session, number of hours of day spent stoned in a session, time of last joint, number of days cannabis was used in the last year, preferred form of cannabis in the last year, and most common method of administration.

Regarding mixing cannabis with tobacco, participants were asked whether they used tobacco mixed with cannabis in the last 12 months, with *Never* and *Yes* provided as response options. Concerning how soon after the participant woke up and had their first joint on the day they used cannabis, the selections of *Immediately within 5 min*, *Within less than an hour*, *Within 1–4 h*, *Within 5–12 h* and *After more than 12 h* were provided. For normal daily cannabis use, participants were asked to select the weight, from a dropdown list of 29 weights, starting at 50 mg and gradually increasing to the final selection of > 20 g. Regarding the number of hours spent stoned in a session, participants were asked to select from a dropdown list of 24 options, increasing in one-hour increments to the last option of *24 Hours*. For the number of days cannabis was used in the last 12 months, participants keyed in their answer in a box provided. Participants were asked how long before bed they had their last joint, with the following options: *Last thing before bed*, *1–2 h before bed*, *3–4 h before bed* and *More than 4 h before bed*. Concerning the most common way participants used cannabis, the following options were provided: *Smoked in a joint (rolled cannabis cigarette) with tobacco*, *Smoked in a joint without tobacco*, *Smoked in a blunt (cigar that has been hollowed out and filled with cannabis) with tobacco, Smoked in a blunt without tobacco, Smoked in a pipe with tobacco, Smoked in a pipe without tobacco, Smoked in a bong/water pipe with tobacco, Smoked in a bong/water pipe (filtration device generally used for smoking cannabis) without tobacco, Bucket bong (method of consuming smokable substances such as cannabis, using two containers), Hot knife (method of smoking cannabis with two knife blades), Vaporizer (device used to vaporize cannabis for inhalation), Eaten in food, Tincture/drank as tea,* and *Medical spray.* For the preferred preparation of cannabis in the last year, participants could select the following: *High potency herbal cannabis*, *Resin/hash (drug made from the resin of the cannabis plant)*, *Normal weed/bush/pressed*, *Edibles (food product that contains cannabinoids)*, *Kief (resinous trichomes of cannabis that may accumulate in containers)*, *Oil*, and *Butane Hash Oil (oil extracted from cannabis using butane as a solvent)*. Prior to analysis, the variable regarding the time of first joint in a day was categorized into *>60mins* and *< 60mins* of waking*,* to model time to first cigarette. The variable regarding the grams of cannabis used per session was recoded into a continuous variable, and the > 20 g value was recoded as 21 g. On average, there are about 0.32 g of cannabis in a joint (Ridgeway & Kilmer, 2016). For ease of interpretation, the age variable was recoded into a categorical variable with intervals of ten years each, and consecutive age groups representing less than 5% of the sample were subsumed into a larger group (41–79 years) for clearer interpretation.

## Results

### Sample

A total of 10,183 respondents from the US completed the survey between November 2016 and January 2017. Of these respondents, 8345 (82%) participants reported cannabis use in the past year. There was missing data on some variables, and we have indicated the total number of cases for each variable (see Table 1). Males accounted for 75.48% of the sample, with a median age of 23 (interquartile range (IQR): 19–32, Range: 16–79, see Table 1).

**Table 1:** Descriptive variables (number of participants reporting cannabis use in last year = 8345)

Age (*N* = 8345)	−16-20	28.4%
	−21-30	41.7%
	−31-40	14.9%
	−41-79	15.0%
Sex (N = 8345)	-Male	75.5%
	-Female	23.6%
	-Transgender	0.9%
Time of First Joint (*N* = 7033)	>60mins	78.0%
	<60mins	22.0%
Time of Last Joint (*N* = 7034)	-Last Thing before Bed	31.3%
	−1-2 h before bed	49.1%
	−3-4 h before bed	15.4%
	-More than 4 h before bed	4.2%
Mixing Tobacco with Cannabis (N = 8345)	No	78.0%
	Yes	22.0%
Cannabis Used Per Session (Grams) (*N* = 7667)	Median	0.5
	Interquartile Range	0.125–1.000
Number of Hours Stoned in a Session (*N* = 6970)	Median	4
	Interquartile Range	3.0–6.0
Number of Days Cannabis was Used in the Last Year (*N* = 7389)	Median	250
	Interquartile Range	50–360
Preferred Form of Cannabis (*N* = 7565)	-High potency herbal cannabis	62.1%
	-Resin/hash	11.2%
	-Normal weed/bush/pressed	1.7%
	-Edibles	1.3%
	-Kief	8.3%
	-Oil	8.0%
	-Butane Hash Oil	7.6%
Most Common Mode of Cannabis Consumption (*N* = 7913)	-Smoked in a joint with tobacco	3.8%
	-Smoked in a joint without tobacco	11.3%
	-Smoked in a blunt with tobacco	0.7%
	-Smoked in a blunt without tobacco	7.5%
	-Smoked in a pipe with tobacco	0.5%
	-Smoked in a pipe without tobacco	33.3%
	-Smoked in a bong/water pipe with tobacco	2.6%
	-Smoked in a bong/water pipe without tobacco	23.0%
	-Bucket bong	1.5%
	-Hot knife	0.2%
	-Vaporizer	12.7%
	-Eaten in food	2.4%
	-Tincture/drank as tea	0.4%
	-Medical spray	0.1%

### Characteristics of Cannabis use

Of those who reported cannabis use in the past year, most (78%) reported consuming their first joint more than an hour after waking, and about half the sample had their last joint 1–2 h before bed (49%) (see Table 1). The majority (78%) tended not to mix tobacco with cannabis. Respondents reported using cannabis for a median of 250 days in the last year (almost daily), with 0.500 g the median per session. Respondents spent a median of four hours a day stoned when cannabis was used. Most (62%) of respondents reported high potency herbal cannabis as their preferred cannabis preparation in the last year, followed by resin/hash (11%). About a third (33%) of participants smoked cannabis in a pipe without tobacco, followed by (23%) smoking it in a bong/water pipe without tobacco.

## Discussion

We sought to provide a descriptive report on cannabis use among a large sample of US-based survey respondents, largely young men, including time of first and last joint, mixing cannabis with tobacco, and other patterns of use. A low proportion of respondents used cannabis within the first hour of waking, suggesting limited preference for *waking and baking (Earleywine et al.,* 2016*)* within young males. By avoiding waking and baking, young male cannabis users may possibly mitigate reductions in poor judgement later in the day, a common outcome when drugs are consumed earlier in the day (Earleywine et al., 2016). Factors that affect altered judgment are key for a demographic prone to risky behaviors (Iritani et al., 2007; Kuntsche et al., 2004). The high proportion of respondents stating time of last joint just before bed may indicate cannabis being used as a sleep aid. Thus, interventions to reduce problematic cannabis use may target young male users who indicate sleep issues as a symptom when purchasing legal cannabis. Most participants do not mix cannabis with tobacco, perhaps indicating an awareness of tobacco’s harmful properties (Agrawal et al., 2009) within young men. Recent research has indicated the increasing use of electronic nicotine delivery systems (McMillen et al., 2012; Soneji et al., 2016), and perhaps such developments are associated with low levels of mixing cannabis with tobacco by young men. Most participants smoked cannabis in a pipe without tobacco. There is some evidence that this may not be the safest way to consume cannabis (Van Dam & Earleywine, 2010). Nevertheless, consuming cannabis without tobacco may be safer than the less popular option of combining the product with tobacco (Meier & Hatsukami, 2016), adding to research around lower risk use. Near daily cannabis use was reported by the majority of respondents, a possible health concern not in line with lower risk cannabis use (Fischer et al., 2017) and such patterns of use may be related to growing US cannabis markets. High potency herbal cannabis was the preferred variant, which may be less harmful compared to more potent, but less popular concentrates (Pierre et al., 2016; Raber et al., 2015). High potency herbal cannabis can contain up to 15% THC (Chan et al., 2017), but concentrates can have up to 40% THC content (ElSohly et al., 2016). The factors underpinning such a product preference may aid understandings around the long-term trajectory of US cannabis use, especially within young men. Several studies, primarily utilizing the NSDUH, NESARC and MTF report the prevalence of US cannabis use, CUD and frequency of use, along with demographic associations (Brook et al., 2013; Cerdá et al., 2012; Earleywine et al., 2016; Hasin et al., 2016; Hasin et al., 2017; Hutmacher, 2015; Terry-McElrath et al., 2017). We extend their work by providing granular data around usage practices, such as cannabis product preferences and time of use, primarily in the young male demographic. Such nuanced data on usage preferences is key given the large range of cannabis products and modes of use. In addition, previous studies were conducted prior to the recent legalization of recreational cannabis in several states, and thus we extend past authors’ work by providing recent data possibly more reflective of current practices.

### Limitations

To the best of our knowledge, this study is the largest US-based study detailing cannabis use patterns. This research design has advantages and disadvantages, such as reliability and validity at a population-based level (Barratt et al., 2017; Winstock & Barratt, 2013; Winstock et al., 2011). Online surveys are considered valid and useful when data are scarce, as with the current study. As hidden samples cannot be efficiently analyzed in generalized population-based surveys, comparable probability samples and ethnographic fieldwork may also be necessary to increase the external validity of our sample (Barratt et al., 2015). Also, as we used on online survey of drug users, our sample was skewed toward younger male participants, and may draw more regular cannabis users.

## Conclusion

We provided data on US cannabis use patterns with largely young male participants, in the wake of rapid growing US legal cannabis markets. Overall, respondents engaged in less problematic modes of cannabis consumption, such as in a pipe without tobacco or in a bong without tobacco. However, the sample were stoned almost daily. High potency herbal cannabis was the preferred variant, which may be less harmful compared to more potent, but less popular concentrates. Frequent drug use may not be an issue in itself, but repeated use of any drug may increase risk of health harms, and thus we suggest that future research explore patterns of cannabis use in the changing US market.

## Abbreviations

GDS: Global drug survey
MTF: Monitoring the future
NESARC: National epidemiologic survey on alcohol and related conditions
NSDUH: National survey on drug use and health
US: United States
